# The effect of extracorporeal shock-wave therapy on pain in patients with various tendinopathies: a systematic review and meta-analysis of randomized control trials

**DOI:** 10.1186/s13102-024-00884-8

**Published:** 2024-04-24

**Authors:** Lobat Majidi, Sorour Khateri, Nikta Nikbakht, Yousef Moradi, Mohammad Reza Nikoo

**Affiliations:** 1 Department of Physical Medicine and Rehabilitation, Faculty of Medicine, Hamadan University of Medical Sciences, Hamedan, Iran; 2 Social Determinant of the Health Research Center, Research Institute for Health Development, Kurdistan University of Medical Sciences, Sanandaj, Iran

**Keywords:** Extracorporeal shock Wave Therapy, Pain, Tendinopathies, Evidence synthesis

## Abstract

**Objectives:**

Tendinopathy is a common condition that affects the body’s tendon structures, causing discomfort, restricted movement, and reduced functionality. In this study, we looked at how extracorporeal shock wave therapy (ESWT) affected pain levels in individuals with various forms of tendinopathy around the world.

**Design:**

This study is a comprehensive review and meta-analysis of previously published randomized controlled trials. To gather relevant data, the researchers performed keyword searches in international databases, including PubMed (Medline), Scopus, Web of Sciences, Cochrane Central Register of Controlled Trials (CENTRAL), Research Registers of ongoing trials (ClinicalTrials.gov), as well as Embase. The search was conducted up until March 2023. The quality of the selected articles was assessed using the Cochrane risk-of-bias method for randomized trials (RoB2).

**Results:**

Based on the results of the meta-analysis, which included 45 clinical studies, the use of ESWT was found to have a significant impact on reducing pain in various conditions. The standardized mean difference (SMD) in patients with plantar fasciitis (PF) was reduced by 1.63 (SMD: -1.63, 95% CI: -3.04, -0.21; I2: 77.36%; P heterogeneity: 0.0001). For lateral epicondylitis (LE), the SMD was 0.63 (SMD: -0.63, 95% CI: -1.11, -0.16; I2: 67.50%; P heterogeneity: 0.003). In the case of chronic Achilles tendinopathy, the SMD was 1.38 (SMD: -1.38, 95% CI: -1.66, -1.10; I2: 96.44%; P heterogeneity: 0.0001). Additionally, in individuals with rotator cuff tendinopathy, the SMD for pain reduction was 2.37 units (SMD: -2.37, 95% CI: -3.58, -1.15; I2: 98.46%; P heterogeneity: 0.0001).

**Conclusion:**

This study suggests that ESWT can be a highly effective therapy option for relieving pain in people with tendinopathy. Nonetheless, it is encouraged to make additional recommendations based on high-quality clinical research and more accurate information in order to define the optimal therapeutic options for each type of tendinopathy.

**Supplementary Information:**

The online version contains supplementary material available at 10.1186/s13102-024-00884-8.

## Introduction

Tendons are composed of units of cells and extracellular matrix surrounded by layers of connective tissue. They serve as conduits for the transmission of force between muscles and bones, facilitating movement [1, 2]. Tendinopathy refers to a broad term used to describe conditions involving the tendons, typically characterized by pain, swelling, and impaired function. It encompasses various tendon disorders, including tendinitis (inflammation of the tendon), tendinosis (degeneration of the tendon without inflammation), and other related conditions [2, 3]. Estimating the prevalence of tendinopathies is challenging, although studies suggest that they constitute approximately 30% of musculoskeletal pain cases [4, 5]. Athletes who participate in strenuous activities are especially prone to tendon injuries, which make up almost half of all sports-related ailments [6–8]. Age, gender, level of physical activity, type of exercise, occupation, and coexisting medical conditions affect the prevalence of tendinopathies [9, 10]. Upper extremity tendinopathies are prevalent, injuries to the tendons of the rotator cuff, particularly affecting the supraspinatus, infraspinatus, and subscapularis tendons, are frequently encountered, leading to discomfort, weakness, and restricted range of motion in the shoulder joint [11–13]. Lateral epicondylitis (tennis elbow) (LE), affecting people who play tennis, golf, swim, or baseball [14–16]. Achilles tendinopathy and patellar tendinopathy (PT) are common tendinopathies of the lower extremities [14, 15, 17]. PT results from the overuse of the knee extensor mechanism and is notably prevalent among male athletes engaged in sports characterized by repetitive jumping movements [18, 19]. External factors contributing to tendinopathy encompass inadequate warm-up and cool-down routines, exercising on rigid surfaces, and sudden alterations in exercise intensity. Internal factors involve biomechanical constraints [20, 21].

Physical therapy, nonsteroidal anti-inflammatory drugs (NSAIDs), bracing, extracorporeal shock wave therapy (ESWT), and acupuncture are some of the methods used. The kind of tendinopathy, intensity, length, patient response, and any comorbidities all influence the therapy approach used [22, 23]. Based on the results of previous studies, some mechanisms of ESWT in alleviating the symptoms of tendinopathy, especially pain, are under discussion. It is generally not recommended for acute cases but is suitable when symptoms persist beyond six months or do not respond to alternative treatments [22, 23]. ESWT functions by applying shock waves to the targeted location, inducing micro-disruptions within the tissues. These microtraumas trigger the production of growth factors, including vascular endothelial growth factor (VEGF), facilitating the recruitment of stem cells to the site of injury [24, 25]. Consequently, ESWT stimulates vascular regeneration, angiogenesis, and increased blood flow, all contributing to tissue healing and inflammation reduction. Moreover, ESWT exhibits direct anti-inflammatory actions, aiding in pain relief [25]. In essence, ESWT leverages a combination of microtrauma-induced healing responses, release of growth factors, activation of stem cells, enhanced blood flow, and anti-inflammatory properties to address pain and facilitate tissue repair across various musculoskeletal conditions [24, 25].

To date, several studies have been conducted worldwide to determine the effect of ESWT alone and to compare it with other treatments, but the results of these studies have shown significant differences and conflicting information [26–29]. For example, a clinical trial conducted in 2005 by Porter and colleagues found that corticosteroid injections were more effective and cost-effective than ESWT in treating PF [30]. However, ESWT was shown to be more suitable and effective than corticosteroids in a 2012 clinical trial by Saber and colleagues [31].

Overall, clinical trials specifically investigating the effects of ESWT have consistently demonstrated significant therapeutic benefits, encompassing enhancements in function, improved quality of life, and substantial alleviation of symptoms, notably pain [32–34]. However, it is important to note that the claim that this intervention is effective requires comparison with other treatments. As mentioned above, the results of these trials are contradictory. In addition, ESWT is used in different ways to treat tendinopathies [35, 36]. For instance, ESWT can be administered either radially or focally, and at varying frequencies or intensities. Radial ESWT employs shock waves that propagate outward in a radial pattern, making it suitable for broader areas such as tendon insertions. Conversely, focal ESWT directs shock waves toward specific targets, enabling deeper penetration and precise tissue targeting, typically employed for localized injuries or abnormalities [37, 38]. ESWT devices also emit shock waves with varying frequencies and intensities [37, 39]. Lower frequencies are appropriate for deeper tissue penetration, whereas higher frequencies may be preferable for superficial treatments [37, 39, 40]. Intensity refers to the energy level of the shock waves, where higher intensities induce greater tissue disruption, potentially beneficial for eliminating calcification or fostering tissue regeneration. In contrast, lower intensities are gentler and more appropriate for mitigating tissue damage, particularly in sensitive areas or specific conditions [37, 39, 40]. The impacts of these varied dosages on treating tendinopathy or mitigating outcomes can differ, underscoring the importance of exploring these effects to ascertain the optimal treatment strategy for these patients. Furthermore, the characteristics and attributes of individuals with tendinopathy may also influence the efficacy of ESWT [41]. Variables such as the patient’s body mass index (BMI), age, and gender may be important factors [41]. To begin, higher BMI levels have been associated with elevated tissue depth and density, potentially affecting shock wave transmission and penetration depth during ESWT. Consequently, this may result in divergent treatment responses among patients with varying BMI levels [42]. Secondly, age-related alterations in tissue composition, vascularity, and healing capacity may modify the responsiveness to ESWT [42–45]. Older patients may have diminished tissue elasticity and blood flow, which might impact shock wave delivery and absorption. Finally, gender differences in tendon structure, hormonal variables, and pain perception may influence therapy success [43–45]. For example, women have been shown to have a higher prevalence of certain tendinopathies and may respond differently to ESWT than men. Therefore, it is critical to examine these patient-specific characteristics [43–45]. Several clinical studies have been conducted worldwide to determine the effect of ESWT on the treatment and improvement of symptoms in patients with tendinopathy [46–50]. However, important aspects such as assessing the effect of ESWT based on radial or focal application, different frequencies or intensities, patient background variables, and comparing results with other treatments have not been adequately addressed. In addition, systematic reviews and meta-analyses have been conducted to determine the effect of ESWT on the treatment and improvement of tendinopathy symptoms worldwide [51–54]. However, these studies have not yielded significant results in terms of methodology, publication date, comparison of effects based on important variables, or comparison with other treatments. An essential outcome related to tendinopathy associated with the use of ESWT is the level of pain resulting from tendinopathy and its reduction after ESWT [55, 56]. Various tools have been used to determine average pain after ESWT, but most published studies have used visual analogue scales or similar instruments. This meta-analysis aims to determine the effect of ESWT, taking into account various factors, on pain levels resulting from different tendinopathies (chronic Achilles tendinopathy (CAT), Achill tendinopathy, plantar fasciitis (PF), chronic proximal plantar fasciitis, conventional treatment, lateral epicondylitis (LE), rotator cuff (RC) tendinopathy, patellar tendinopathy (PT)) in order to provide valuable insights for optimizing treatment approaches.

## Methods

This systematic review and meta-analysis were conducted in accordance with the Preferred Reporting Items for Systematic Reviews and Meta-Analyses (PRISMA) guidelines, which provide recommendations for the design, conduct, and reporting of systematic reviews and meta-analyses [57]. The protocol for this study was registered in Prospero under the registration number CRD42022334221. The Ethics Committee of Hamedan University of Medical Sciences, Hamedan, Iran, approved this study (IRB: IR.UMSHA.REC.1401.715).

### Comprehensive search strategy

Two independent researchers (SKH and LM) conducted a comprehensive search of randomized controlled trials in major international databases, including Medline (PubMed), Web of Science, Scopus, the Cochrane Central Register of Controlled Trials (CENTRAL), and the research registers of ongoing trials (ClinicalTrials.gov). The search encompassed the period from 1990 to the end of March 2023. Relevant keywords and English phrases, namely “extracorporeal shock wave therapies,” “pain,” “plantar fasciitis,” “Achilles tendinopathy,” “rotator cuff tendinopathy,” and “lateral epicondylitis,” were utilized to identify eligible studies (Table 1). The search results were imported into EndNote version 8, and duplicate studies were meticulously excluded. Subsequently, the initial search results underwent a rigorous screening process based on predefined inclusion and exclusion criteria.

**Table 1:** Search syntax

PubMed (((“Extracorporeal Shockwave Therapies“[All Fields] OR “Extracorporeal Shockwave“[All Fields] OR “Shockwave“[All Fields]) AND (“fasciitis, plantar“[MeSH Terms] OR (“fasciitis“[All Fields] AND “plantar“[All Fields]) OR “plantar fasciitis“[All Fields] OR (“plantar“[All Fields] AND “fasciitis“[All Fields]))) OR “Achilles Tendinopathy“[All Fields] OR “Patellar“[All Fields] OR “Rotator Cuff Tendinopathy“[All Fields] OR “Lateral Epicondylitis“[All Fields]) AND (“pain“[MeSH Terms] OR “pain“[All Fields]) Scopus (TITLE-ABS-KEY (“Extracorporeal Shockwave Therapies”) OR TITLE-ABS-KEY (“Extracorporeal Shockwave”) OR TITLE-ABS-KEY (shockwave) AND TITLE-ABS-KEY (“Plantar Fasciitis”) OR TITLE-ABS-KEY (“Achilles Tendinopathy”) OR TITLE-ABS-KEY (“Rotator Cuff Tendinopathy”) OR TITLE-ABS-KEY (“Lateral Epicondylitis”) OR TITLE-ABS-KEY (“Patellar”) AND TITLE-ABS-KEY (pain)) Web of Sciences 1# TOPIC: (Shockwave) OR TOPIC: (“Extracorporeal Shockwave Therapies”) OR TOPIC: (“Extracorporeal Shockwave”) 2#TOPIC: (“Plantar Fasciitis”) OR TOPIC: (“Achilles Tendinopathy”) OR TOPIC: (“Rotator Cuff Tendinopathy”) OR TOPIC: (“Lateral Epicondylitis”) OR TOPIC: (“Patellar”) 3# TOPIC: (pain) #3 AND #2 AND #1	

### Inclusion and exclusion criteria

The primary objective of this study was to evaluate the impact of ESWT on pain levels among patients diagnosed with common tendinopathies, specifically PF (that affects the tendons of the lower extremities), Achilles tendinopathy, LE, and RC tendinopathy. Initially, clinical trials were considered for inclusion if they involved two groups: one group receiving various forms of ESWT (radial or focal) and another group receiving alternative treatments without ESWT. The selection criteria were based on the PICOT framework (Table 2), which required the inclusion of studies that involved patients with different types of tendinopathies, employed ESWT as an intervention, and utilized the visual analogue scale (VAS) with a range of 0 to 10 to measure the primary outcome of mean pain levels before and after the intervention. The VAS is commonly used in numerous studies and clinical trials, including those involving ESWT, due to its simplicity, sensitivity, and reproducibility [58–61]. Studies that did not meet the criteria of clinical trials, such as cohort studies, case-control studies, case reports, letters to the editor, reviews, and books, were excluded from the meta-analysis. Moreover, non-clinical studies that measured average pain using indicators other than the VAS score or compared the intervention group with treatments other than ESWT were also excluded. Animal and non-human laboratory studies were not considered for analysis.

**Table 2:** PICOT structure

Population (P)	Intervention (I)	Comparison (C)	Outcomes (O)	Type of studies (T)
Patients with various types of tendinopathy like chronic Achilles tendinopathy (CAT), Achill tendinopathy, plantar fasciitis (PF), chronic proximal plantar fasciitis, conventional treatment, lateral epicondylitis (LE), rotator cuff tendinopathy (RC tendinopathy), patellar tendinopathy (PT).	Extracorporeal shock wave therapy (ESWT); focused shock wave (FoSW) and radial shock wave (RaSW).	Other treatments excluding ESWT	The mean pain measured by the visual analogue scale (VAS) score	Randomized Control Trials (RCTs)

### Screening and final article selection

After the completion of the search and the importation of articles into EndNote version 8, a screening process was conducted based on the title, abstract, and full text of the articles. Initially, two authors independently (SKH and LM) screened the articles based on the topic. Subsequently, the screening process proceeded with the evaluation of the abstracts, followed by a thorough assessment of the full text articles. Both authors independently applied the predefined inclusion and exclusion criteria during each stage of the screening process. In situations where there was a discrepancy or disagreement between the authors (YM), consultation with an expert in the field was sought to reach a consensus. The final selection of articles for inclusion was made by the authors after reviewing the full text or the final versions of the articles.

### Data extraction

After evaluation of titles, abstracts and texts, the full text of selected articles was analyzed in detail. Extraction was performed using a data collection form that included items such as first author’s name, publication date, study type, geographical region, sample size, type and duration of intervention, type of comparison group, average pain based on VAS score at different weeks, BMI and patient age. The entire process, from systematic search to final data extraction, was performed independently by two trained authors (SKH and NN). Any discrepancies were assessed by both authors, and in case of disagreement, the supervising expert was consulted (YM).

### Quality assessment of articles (risk of bias)

The risk of bias in the included studies was assessed using the Cochrane risk-of-bias tool for randomized trials (RoB 2) [62]. Areas assessed for bias included sequence generation, allocation concealment, blinding, outcome data and outcome reporting. Trials were considered to be at high risk of bias if methodological flaws were likely to affect the true outcome. Trials were considered to be at low risk of bias if the shortcomings were considered to be unimportant for the actual outcome. If there was not enough information to make a judgement, the risk of bias was considered unclear.

### Data synthesis

In this meta-analysis, the statistical analysis was conducted using STATA version 17. Initially, baseline means were calculated, followed by the calculation of the difference of interest between the two groups. In the meta-analysis, the combined means were used, and the primary outcome measure for reporting was the standardized mean difference (SMD) [63]. To assess methodological and statistical heterogeneity, the I-squared index was employed. Clinical heterogeneity was determined through expert opinion. Publication bias was evaluated using a funnel plot and Egger’s test. A significance level below 5% was considered in this study. Subgroup analyses were performed to explore the influence of various variables, including patient age, BMI, duration of follow-up after the intervention, hertz and pulse of the intervention, type of tendinopathy, and number of intervention sessions. These subgroup analyses aimed to identify potential sources of heterogeneity. Additionally, regression analysis was utilized to examine the impact of these variables on the association of interest.

## Results

**Fig. 1:**
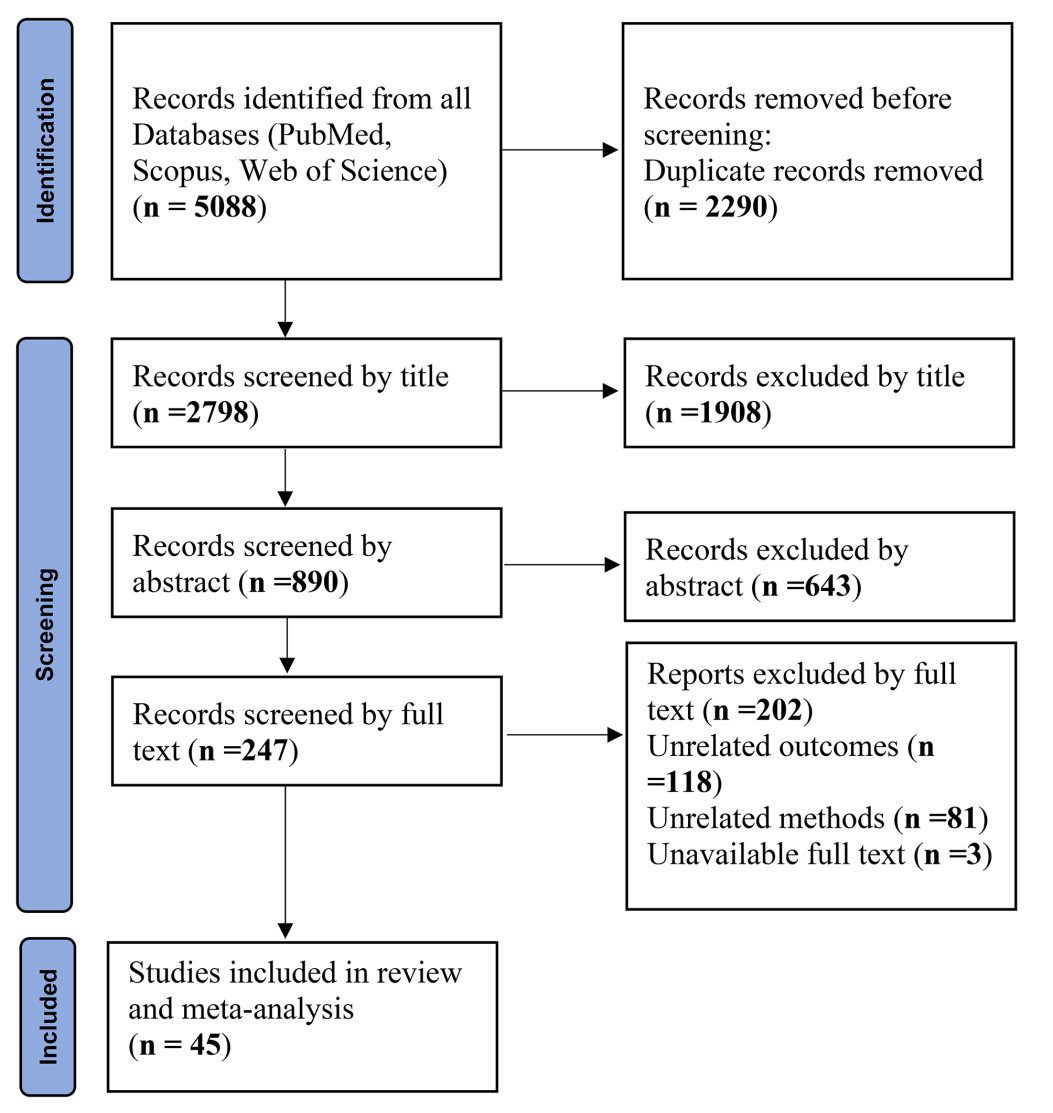
PRISMA 2020 flow diagram included searches of databases and registers only with their results

After conducting a comprehensive search of international databases and retrieving relevant articles based on pre-established inclusion and exclusion criteria, a systematic screening process was undertaken by assessing the topic, abstract, and full text of the identified studies. The detailed results of this stage are presented in Fig. 1. Initially, a total of 5,088 articles were obtained from international databases, with 2,290 identified as duplicates. Subsequently, in the title screening stage, 2,798 articles were reviewed, resulting in the exclusion of 1,908 articles. This left 890 articles for further screening based on their abstracts. Following the abstract screening stage, 643 articles were eliminated, leaving 247 articles for the subsequent full-text screening stage. Upon careful examination of the full text and the application of the predefined study inclusion criteria, 118 articles were excluded due to irrelevant outcomes, while 81 articles were excluded due to inappropriate methodology. Additionally, three articles were excluded due to unavailability of the full text. Finally, a total of 45 studies remained eligible for further analysis (Fig. 1).

Among the studies included in the analysis, all of them were clinical trials and had been published. Specifically, 13 studies focused on the outcome of PF, 3 studies examined the outcome of CAT, 22 studies investigated LE, 3 studies explored RC tendinopathy, and 5 studies examined PT. The most commonly utilized tool for measuring pain across the majority of these selected studies was the VAS, as indicated in Table 3. In terms of the control group employed in these trials, there was considerable variation. Some studies utilized a placebo group for comparison with ESWT, while others employed different pain-relieving methods such as corticosteroid injections (CSI), prolotherapy, conventional treatment, platelet-rich plasma (PRP) or autologous conditioned plasma (ACP), eccentric loading, or exercise. Detailed information regarding these comparisons can be found in Table 3.

**Table 3:** the characteristics of included and selected studies

Authors (Years) (Re)	Country	Study Population (Age), (BMI)	Type of Studies	Type of tendinopathy	Type shockwave (N)	Pulse and Frequency (N sessions)	Comparison group (N)	Assessment outcomes
T. W. Lai, et al. (2018) [64]	China	Patient with PF (54), (NR)	RCT	PF	ESWT (47)	1500 Pulse 1–6 Hz (2)	CSI (50)	4 and 12 weeks
B. Vahdatpour, et al. (2018) [65]	Iran	Patient with CAT (54), (NR)	RCT	Achill tendinopathy	FoSW RaSW (22)	1500–3000 Pulse 2.3 Hz (4)	Sham ESWT (21)	4 and 16 weeks
J. D. Rompe, et al. (2008) [66]	Germany	Insertional CAT (40), (NR)	RCT	Achill tendinopathy	RaSW (25)	2000–4000 Pulse 8 HZ (3)	Eccentric Loading (25)	16 weeks
J. D. Rompe, et al. (2009) [67]	Germany	Midportion CAT (50), (NR)	RCT	Achill tendinopathy	RaSW (34)	2000 Pulse 8 HZ (3)	Eccentric Loading (34)	16 weeks
J. D. Rompe, et al. (2007) [68]	Germany	Main Body Achill tendinopathy (50), (NR)	RCT	Achill tendinopathy	RaSW (25)	2000 Pulse 8 Hz (3)	Eccentric Training (25) Wait and See (25)	16 weeks
Y. Bahar-Ozdemir, et al. (2021) [69]	Turkey	Patient with PF (52), (31)	RCT	PF	FoSW (15)	3000 Pulse 11-Hz (5)	ESWT plus KT (15) ESWT plus Sham KT (14)	4 weeks
S. Hocaoglu et al(2017) [70]	Turkey	Patient with PF (49), (28.5)	RCT	PF	RaSW (36)	2000 Pulse 10-Hz (3)	Radial ESWT (36) Corticosteroid (36)	4, 12, and 24 weeks
M. Bicer, et al. (2018) [71]	Turkey	Patient with PF (45), (29.6)	RCT	PF	ESWT (30)	2500 Pulse 12–15 Hz (3)	KT (33)	1 weeks
B. Ordahan, et al. (2017) [72]	Turkey	Patient with PF (47), (32)	RCT	PF	ESWT (37)	2500 Pulse 12–15 Hz (5)	NR	5 weeks
M. Asheghan, et al. (2021) [73]	Iran	Patient with PF (45), (26)	RCT	Chronic - PF	RaSW (29)	2000 Pulse 10 Hz (3)	Prolotherapy (30)	6 and 12 weeks
M. Rahbar, et al. (2018) [74]	Iran	Patient with PF (44), (26)	RCT	PF	ESWT (36)	2000 Pulse 10 Hz (3)	Dry needling (36)	4 and 8 weeks
D. S. Hammer, et al. (2002) [75]	Germany	Patient with CPPF (45), (29.6)	RCT	Chronic - PF	ESWT (24)	3000 Pulse 16–20 Hz (3)	Conventional treatment (24)	6, 12, and 24 weeks
L. Gerdesmeyer, et al. (2017) [76]	Germany	Patient with PF (51), (28)	RCT	PF	ESWT (52)	2000 Pulse 8 Hz (1)	Placebo (53)	6 weeks
C. Razzano, et al. (2017) [77]	Italy	Patient with PF (51), (26)	RCT	PF	ESWT (49)	2000 Pulse (3)	NIN (55)	4 and 12 weeks
K. T. L. Chew, et al. (2013) [78]	Singapore	Patient with PF (46), (25)	RCT	PF	ESWT (19)	2000 Pulse (42)	ACP (19) Conventional treatment (16)	4, 12 and 24 weeks
F. Eslamian, et al. (2016) [79]	Iran	Patient with PF (41), (NR)	RCT	PF	RaSW (20)	2000 Pulse 2- Hz (5)	CI (20)	4 and 8 weeks
M. Corum, et al(2021) [80]	Turkey	patients with LE (46), (25.6)	RCT	LE	RaSW (20)	2000 Pulse 10 Hz (3)	Exercise group (19)	4 and 12 weeks
B. Chung, et al. (2005) [81]	Canada	Patients with LE (46), (26.5)	RCT	LE	ESWT (31)	2000 Pulse 10 Hz (3)	Sham ESWT (29)	4, 8 and 12 weeks
M. S. Beyaza et al. (2015) [82]	Turkey	Patients with LE (40), (29)	RCT	LE	RaSW (32)	2000 Pulse 16 Hz (3)	CSI (32)	4 and 12 weeks
M. C. Vulpiani, et al. (2015) [83]	Italy	Patients with chronic LE (51.5), (NR)	RCT	Chronic– LE	FoSW (40)	2400 Pulse (NR)Hz (3)	Cryo-US (40)	12, 24, and 48 Weeks
J. D. Rompe, et al. (1996) [84]	Germany	Patients with Chronic LE (42.9), (NR)	RCT	Chronic– LE	ESWT (50)	3000 Pulse (3)	Control (50)	3, 6, and 24 Weeks
M. P. Staples, et al. (2008) [85]	Australia	patients with chronic LE (49.5), (NR)	RCT	Chronic– LE	ESWT (28)	2000 Pulse (3)	Placebo (27)	6, 12, and 24 Weeks
T. Guler, et al. (2020) [86]	Turkey	Patients with LE (42.5), (NR)	RCT	Acute– LE	ESWT (20)	2000 Pulse (3)	KT (20)	4, and 8 Weeks
J. D. Rompe, et al. (2004) [87]	Germany	Patient with chronic LE (45), (22.8)	RCT	Chronic– LE	ESWT (34)	2000 Pulse 4 Hz (3)	Sham ESWT (36)	12 and 48 weeks
C. G. Aydin, et al. (2017) [88]	Turkey	Patient with LE (46), (NR)	RCT	LE	ESWT (27)	1200 Pulse 5 Hz, (4)	Placebo (19)	80 weeks
B. Vahdatpour, et al. (2020) [89]	Iran	Patient with LE (40), (NR)	RCT	LE	ESWT (35)	2000 Pulse 5 Hz (3)	Placebo (35)	4 and 8 weeks
B. Yalvac, et al. (2018) [90]	Turkey	Patient with LE (44), (NR)	RCT	LE	ESWT (20)	2000 Pulse 10–15 Hz (3)	Ultrasound (24)	1 and 4 weeks
E. D. Collins, et al. (2011) [91]	USA	Patient with chronic LE (45.7), (NR)	RCT	Chronic– LE	ESWT (90)	1500 Pulse (1)	Placebo (91)	4, 8, 12, 24 and 48 weeks
B. Sarkar, et al. (2013) [92]	India	Patient with chronic LE (41), (NR)	RCT	Chronic– LE	ESWT (15)	2000 Pulse (3)	Exercise (15)	4 weeks
C. A. Speed, et al. (2002) [93]	UK	Patient with LE (48), (NR)	RCT	LE	ESWT (40)	1500 Pulse (3)	Sham ESWT (35)	4, 8 and 12 weeks
P. Lizis, et al. (2015) [94]	Poland	Patient with chronic LE (48.5), (NR)	RCT	LE	ESWT (25)	1500 Pulse 8 Hz (5)	Ultrasound (25)	1 and 12 weeks
N. Capan, et al. (2016) [95]	Turkey	Patient with LE (48), (27.5)	RCT	LE	RaSW (28)	2000 Pulse 10 Hz (3)	Placebo (28)	4 and 12 weeks
T. H. Yang, et al. (2017) [96]	Taiwan	Patient with LE (50), (25.9)	RCT	LE	RaSW (15)	2000 Pulse 10 Hz (3)	Placebo (13)	6, 12, 24 weeks
D. Celik, et al. (2019) [97]	Turkey	Patient with LE (48), (NR)	RCT	LE	FoSW (20)	2000 Pulse 10 Hz (4)	Photo biomodulation therapy (23)	1 and 12 weeks
T. Ahadi, et al. (2019) [98]	Iran	Patient with LE (47), (NR)	RCT	LE	ESWT (16)	2000 Pulse 10 Hz (3)	Prolotherapy (17)	4 and 8 weeks
A. Aydin, et al. (2018) [99]	Turkey	Patient with LE (38), (27)	RCT	LE	ESWT (32)	2000 Pulse 10–12 Hz (4)	WESs (35)	4, 12 and 24 weeks
T. Ozmen, et al. (2021) [100]	Turkey	Patient with LE (47.7), (28.5)	RCT	LE	ESWT (14)	1500 Pulse 4 Hz (3)	Kinesio taping (13)	2 and 8 weeks
T. Ozmen, et al. (2021) [100]	Turkey	Patient with LE (49), (27.5)	RCT	LE	ESWT (14)	1500 Pulse 4 Hz (3)	Ultrasound (13)	2 and 8 weeks
J. Y. Ko, et al. (2020) [101]	Taiwan	Patients with RC tendinopathy (51.5), (23.8)	RCT	RC tendinopathy	ESWT (16)	3000 Pulse (1)	Sham (15)	1, 4, 12, 24 and 48 weeks
K. Engebretsen, et al. (2011) [102]	Norway	Patients with subacromial shoulder pain (48), (NR)	RCT	RC tendinopathy	RaSW (52)	2000 Pulse 8–12 Hz (4–6)	Supervised exercises (52)	6, 12, 18, and 48 weeks
V. Dedes, et al. (2019) [103]	Greece	Patients with RC tendinopathy (NR), (NR)	RCT	RC tendinopathy	RaSW (56)	2000 pulse 15 Hz (4–6)	Ultrasound (47)	1 and 4 weeks
V. Dedes, et al. (2019) [103]	Greece	Patients with RC tendinopathy (NR), (NR)	RCT	RC tendinopathy	RaSW (56)	2000 pulse (15) Hz (4–6)	Control (12)	1 and 4 weeks
J. Zwerver, et al. (2010) [104]	Netherlands	Athletes with mild chronic PT (NR), (NR)	RCT	Knee (PT)	FoSW (31)	NR	Placebo (31)	1, 12, and 22 Weeks
L. Cheng, et al. (2019) [105]	China	Athletes with PT (22.3), (NR)	RCT	Knee (PT)	ESWT (26)	2000 Pulse (9–12) Hz (16)	Physical treatments: (acupuncture, ultrasonic wave, microwave therapy) (25)	16 Weeks
K. M. Thijs, et al. (2017) [106]	Netherlands	Athletes with PT (28.6), (23.6)	RCT	Knee (PT)	FoSW (22)	1000 Pulse 4 Hz (3)	Placebo (30)	6, 12, and 24 weeks
M. Vetrano, et al. (2013) [107]	Italy	Athletes with jumper’s knee (26.9), (NR)	RCT	Knee (PT)	FoSW (23)	2.400 Pulse (3)	PRP Injection (23)	8, 24, and 48 weeks
Z. J. Zhang, et al. (2020) [108]	China	Athletes with PT (22.2), (23)	RCT	Knee (PT)	FoSW (17)	1500 Pulse 4 Hz (1)	Sham (17)	Post treatment

CAT: chronic Achilles tendinopathy, CSI: corticosteroids injections, NRSS: numerical rating scale scores, PF: plantar fasciitis, KT: Kinesio taping, FoSW: Focused ESWT, RaSW: Radial ESWT, ESWT: extracorporeal shock wave therapy, NIN: noninvasive interactive neurostimulation, ACP: Autologous conditioned plasma; PRP: Platelet-rich plasma, LE: Lateral epicondylitis, Cryo-US: Cry- ultrasound, PT: Patellar tendinopathy, WES: Wrist extensor splint; RCT: Randomized controlled trials

### Mean pain in patients with PF

**Fig. 2:**
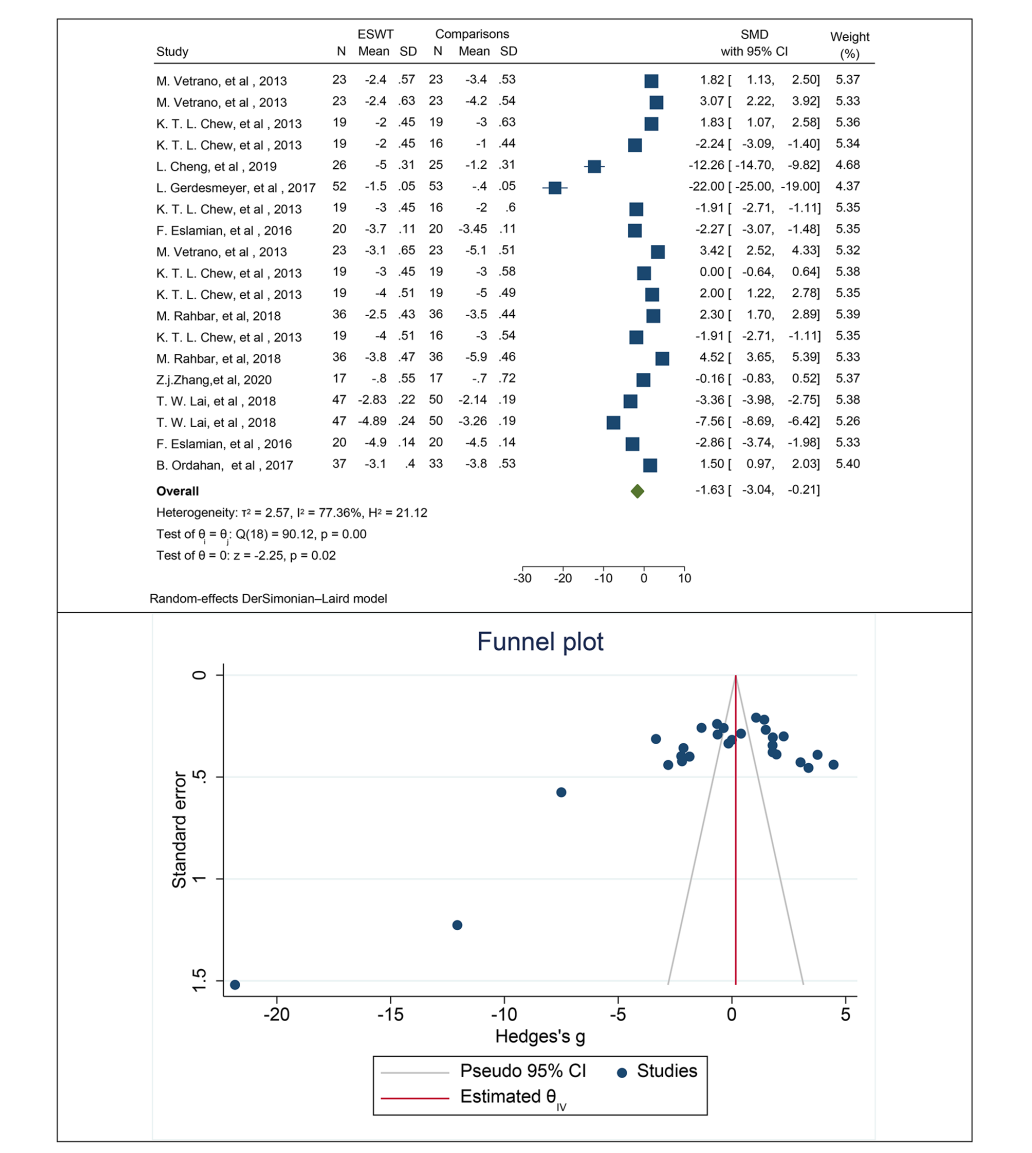
The forest plot displays the pooled standardized mean difference (SMD) in pain among patients with plantar fasciitis

Among the studies selected for this systematic review and meta-analysis, 13 studies specifically focused on evaluating the outcome of PF. Out of these 13 studies, 9 studies reported the mean pain in two groups based on the VAS score, while 4 studies reported the mean pain as a percentage using the same measurement tool. Pooling the data from these studies to estimate the overall effect of ESWT on mean pain in patients with PF, the results indicated a significant reduction in mean pain based on the VAS score, with a SMD of -1.63 (95% CI: -3.04, -0.21; I2: 77.36%; P heterogeneity: 0.0001) (Fig. 2). To assess publication bias, Egger’s test was employed, which revealed the presence of publication bias (B: -17.31; SE: 2.29; P: 0.0001). Additionally, publication bias was examined using a funnel plot, as depicted in Fig. 2. In order to account for the potential impact of this bias on the overall estimated mean, a trim and fill analysis was conducted. This analysis indicated that the mean, after adjusting for bias, was − 1.62, which was not significantly different from the reported cumulative estimate of -1.63.

A subgroup analysis was conducted to examine the impact of patient age on the relationship between the use of ESWT and mean pain, as measured by the crude mean VAS score. The results revealed that with each year increase in patient age, ESWT was associated with a reduction in mean pain by 3 units (B: -3.68; SE: 1.56; 95% CI: -10.67, -1.22; *P* value: 0.003). Additionally, subgroup analyses were performed based on several factors, including the number of ESWT sessions, frequency and pulse intensity of the intervention, duration of patient follow-up after the intervention, and type of comparison group in the selected studies. The findings of these subgroup analyses are presented in Table 4. The results demonstrated that, in terms of age and BMI, ESWT significantly reduced mean pain in patients with PF when their age exceeded 30 years (SMD age > 30: -2.02, 95% CI: -3.69, -0.35; I2: 87.49%; P heterogeneity: 0.0001) and when their BMI was higher than 25 (SMD BMI > 25: -2.80, 95% CI: -6.64, -0.05; I2: 58.95%; P heterogeneity: 0.0001). Furthermore, a subgroup analysis based on the number of ESWT sessions indicated that the effect of ESWT in reducing mean pain was more pronounced when the treatment consisted of five or more sessions for patients with PF (SMD: -3.75, 95% CI: -7.21, -0.28; I2: 47.97%; P heterogeneity: 0.098) (Table 4).

**Table 4:** Subgroup analyses related to determining the effect of ESWT on mean pain in patients with plantar fasciitis

Outcomes	Variables for subgroups	No. of effect size	Pooled standardized mean difference (SMD) (% 95 CI)	Heterogeneity assessment (within groups)			Heterogeneity assessment (within groups)	
				I square	Q test	*P* value	Q test	*P* value
PF	Age							
	≤ 30	5	-0.50 (-3.11, -0.11)	78.73%	51.96	0.0001		
	> 30	14	-2.02 (-3.69, -0.35)	87.49%	66.09	0.0001	0.92	0.340
	BMI							
	≤ 25	7	-0.34 (-1.58, 0.91)	34.82%	12.76	0.099		
	> 25	4	-2.80 (-6.64, -0.05)	58.95%	55.65	0.048	1.43	0.238
	Sessions							
	< 5 weeks	15	-1.09 (-2.72, -0.53)	18.44%	5.9	0.652		
	≥ 5 weeks	4	-3.75 (-7.21, -0.28)	47.97%	15.33	0.098	1.85	0.170
	HZ							
	< 10	6	-5.93 (-8.65, -3.20)	68.27%	21.76	0.08		
	≥ 10	4	-0.58 (-3.51, -0.06)	70.21%	27.45	0.088	6.86	0.010
	Plus							
	≤ 2000	15	-2.77 (-4.45, -1.08)	77.01%	45.49	0.001		
	< 2000	4	2.40 (1.50, 3.30)	70.43%	38	0.005	28.08	0.0001
	Follow up							
	5–10 weeks	7	-0.34 (-2.07, -0.01)	97.88%	82.37	0.001		
	10–15 weeks	4	-4.33 (-9.72, -1.06)	99.81%	66.5	0.001	3.19	0.365
	15–20 weeks	3	-3.13 (-6.99, -0.73)	98.46%	29.58	0.001		
	> 20	5	-0.91 (-4.05, -0.23)	98.81%	19.92	0.001		
	Type of ESWT							
	RaSW	7	-0.26 (-1.96, -0.01)	99.60%	88.31	0.001		
	FoSW	4	-1.98 (-3.55, -0.42)	94.16%	41.5	0.001	7.47	0.029
	Not determined	18	-2.06 (-4.84, -0.73)	98.19%	30.02	0.001		
	Type of comparisons							
	Placebo	2	-11.03 (-32.43, -4.38)	33.48%	5.31	0.077		
	CSI	5	-5.43 (-9.54, -1.32)	0.00%	3.18	0.87	103.2	0.0001
	ACP	3	1.26 (-0.06, 2.58)	90.08%	40.39	0.004		
	PRP	3	2.74 (1.72, 3.75)	78.52%	20.16	0.089		
	Others	4	-0.58 (-3.51, 2.36)	58.21%	8.57	0.087		

CSI: corticosteroids injections, PF: plantar fasciitis, FoSW: Focused ESWT, RaSW: Radial ESWT, ESWT: Extracorporeal shock wave therapy, Autologous conditioned plasma: ACP; Platelet-rich plasma: PRP, Cryo-US: Cry- ultrasound, WES: Wrist extensor splint; RCT: Randomized controlled trials; CI: Confidence interval

In addition, ESWT has a significant effect on reducing patients’ mean pain when the frequency is less than 10 Hz (SMD HZ < 10: -5.93, 95% CI: -8.65, -3.20; I2: 68.27%; P heterogeneity: 0.080) and the pulse is less than 2000 (SMD Plus < 10: -2.77, 95% CI: -4.45, -1.08; I2: 77.01%; P heterogeneity: 0.001) (Table 4).

Based on the classification of studies according to the type of ESWT utilized, the studies were grouped into three categories: radial (RaSW), focused (FoSW), and not determined. The results indicated that the effect of ESWT on pain reduction in patients with PF differed across these categories. For RaSW, the mean pain reduction was 0.26 units (SMD: -0.26, 95% CI: -1.96, -0.01; I2: 99.60%; P heterogeneity: 0.001). In the case of FoSW, the mean pain reduction was approximately 2 units (SMD: -1.98, 95% CI: -3.55, -0.42; I2: 94.16%; P heterogeneity: 0.001) (Table 4). Subgroup analyses were also conducted based on the type of comparison group used in relation to ESWT. These groups included a placebo group, CSI, ACP, and PRP. The findings revealed that the effect of ESWT in reducing mean pain was more significant in the placebo group compared to the other comparison groups. ESWT resulted in an average pain reduction of 11.03 units compared to placebo (SMD: -11.03, 95% CI: -32.43, -4.38; I2: 33.48%; P heterogeneity: 0.077). In contrast, the reduction in mean pain was 5.43 units for the CSI group (SMD: -5.43, 95% CI: -9.54, -1.32; I2: 77.52%; P heterogeneity: 0.044). Notably, the effect of ESWT on ACP or PRP did not lead to a reduction in mean pain among patients with PF (Table 4).

### Mean pain in patients with LE

**Fig. 3:**
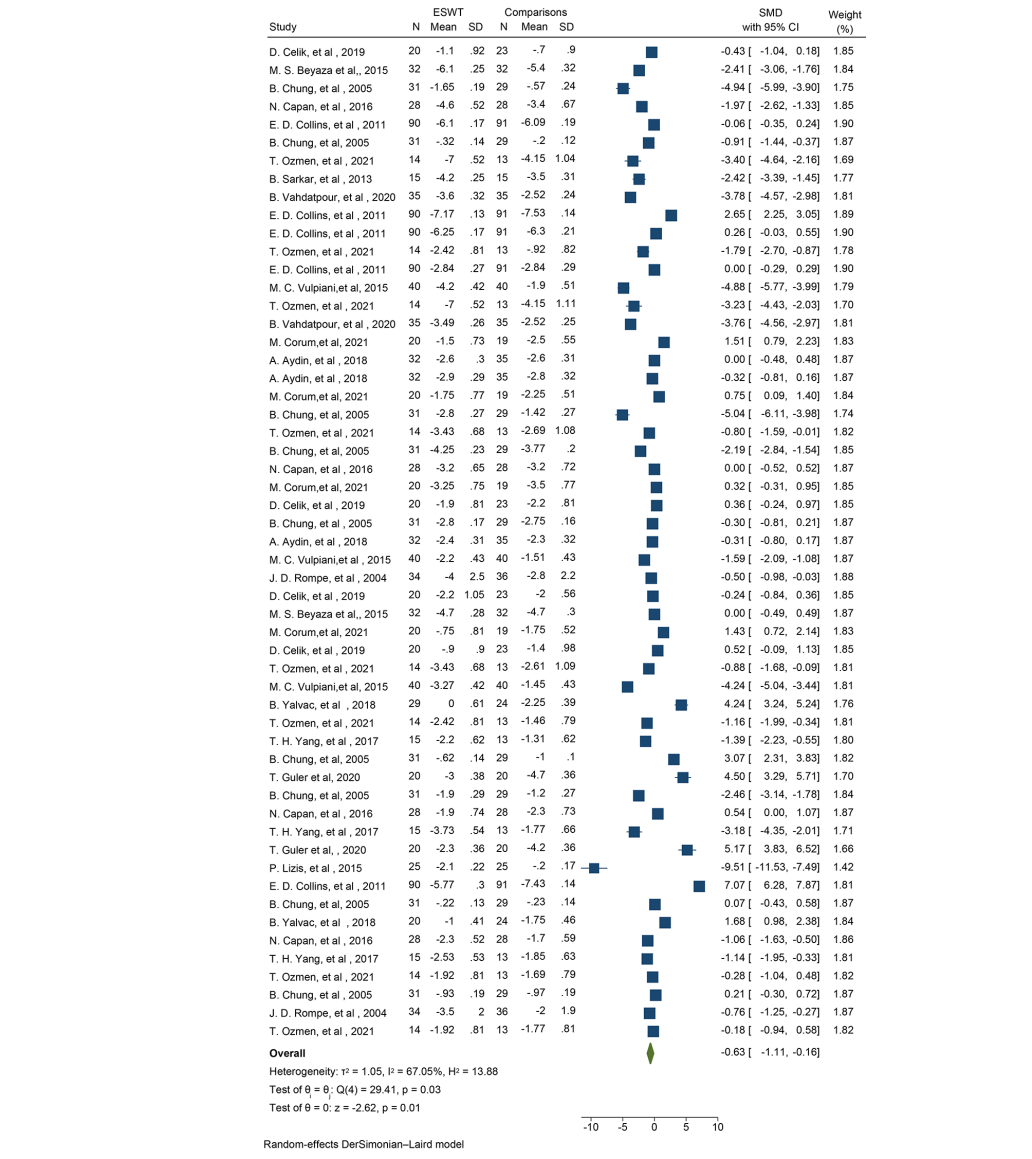
The forest plot displays the pooled standardized mean difference (SMD) for pain among patients with LE

Among the selected studies, 22 specifically addressed the outcome of LE. Out of these 22 studies, 16 trials (with 55 reported mean differences) reported the mean pain in two groups using the crude VAS score, while 6 trials (with 18 reported mean differences) reported the mean pain as a percentage using the same measurement tool. Pooling the data from these studies to estimate the overall effect of ESWT on the mean VAS score in patients with LE, the results showed a significant reduction in mean pain based on the VAS score, with an average reduction of 0.63 units (SMD: -0.63, 95% CI: -1.11, -0.16; I2: 67.50%; P heterogeneity: 0.003) (Fig. 3). To evaluate publication bias, Egger’s test was employed and indicated the presence of publication bias (B: -6.31; SE: 2.42; P: 0.009). To account for the potential impact of this bias on the overall calculated mean, a trim and fill analysis was conducted. The analysis revealed that the mean, after adjusting for bias, was − 0.65, which was not significantly different from the reported cumulative estimate of -0.63.

Subgroup analyses were conducted to examine the impact of various factors on the effect of ESWT on mean pain scores in patients with LE. The analyses considered patient age, BMI, number of ESWT sessions, pulse intensity and frequency of the intervention, duration of patient follow-up after the intervention, and the type of comparison group in the selected studies. The results of the subgroup analyses indicated that the effect of ESWT on mean pain scores in patients with LE increased with higher patient age and BMI. Additionally, the effect of ESWT was found to be greater in patients with chronic LE compared to other patients (SMD: -1.02; 95% CI: -2.22, -0.18; I2: 89.72%; P heterogeneity: 0.0001). Regarding the number of ESWT sessions, pulse intensity, and frequency, the subgroup analyses suggested that a higher number of sessions (more than 5 sessions) (SMD: -1.12; 95% CI: -4.16, -0.02; I2: 86.99%; P heterogeneity: 0.0001), low frequency below 15 (SMD: -1.38; 95% CI: -2.06, -0.70; I2: 41.49%; P heterogeneity: 0.097), and high pulse intensity above 2000 (SMD: -3.54; 95% CI: -5.54, -1.55; I2: 79.59%; P heterogeneity: 0.79–5.59) were associated with a greater and more significant reduction in the crude mean pain score.

Furthermore, the results suggested that longer follow-up durations should be considered for these interventions to assess their sustained effects on mean pain scores in patients with LE (Table 5). The results showed that the effect of ESWT on pain in patients with LE decreases by an average of 0.49 when the type of ESWT is RaSW (SMD: -0.49, 95% CI: -1.27, -0.09; I2: 88.99%; P heterogeneity: 0.001). In the case of FoSW type, the average pain decreases by nearly 1.48 units (SMD: -1.48, 95% CI: -3.10, -0.14; I2: 87.03%; P heterogeneity: 0.001) (Table 5). Based on different comparison groups, the results also showed that the effect of ESWT is different compared to different comparison groups such as US, CI, exercise, KT, etc. Compared to US (SMD: -1.94; 95% CI: -4.24, -0.36; I2: 88.79%; P heterogeneity: 0.001) and CI (SMD: -1.19; 95% CI: -3.55, -0.38; I2: 77.12%; P heterogeneity: 0.001), ESWT showed a greater effect (Table 5).

**Table 5:** Subgroup analyses related to determining the effect of ESWT on mean pain in patients with Lateral Epicondylitis (Tennis Elbow)

Outcomes	Variables for subgroups	No. of effect size	Pooled standardized mean difference (SMD) (% 95 CI)	Heterogeneity assessment (within groups)			Heterogeneity assessment (within groups)	
				I square	Q test	*P* value	Q test	*P* value
LE	Age							
	≤ 45	14	0.04 (-0.96, 1.03)	79.80%	44.75	0.0001		
	> 45	41	-0.86 (-1.41, -0.31)	79.01%	38.22	0.0001	2.47	0.120
	BMI							
	≤ 27	21	-0.72 (-1.32, -0.12)	59.90%	47.68	0.0001		
	> 27	14	-1.12 (-1.69, -0.54)	68.37%	22.47	0.0001	2.47	0.250
	Type of LE							
	Chronic	12	-1.02 (-2.22, -0.18)	89.72%	59.84	0.0001		
	Non- chronic	43	-0.54 (-1.02, -0.05)	55.55%	24.53	0.0001	0.54	0.460
	Sessions							
	< 5 weeks	49	-0.76 (-1.39, -0.14)	79.97%	66.81	0.0001		
	≥ 5 weeks	6	-1.12 (-4.16, -0.02)	86.99%	87.32	0.0001	0.16	0.001
	HZ							
	< 15	45	-1.38 (-2.06, -0.70)	41.49%	16.09	0.097		
	≥ 15	9	1.35 (-0.36, 3.07)	68.49%	69.91	0.0001	8.45	0.0001
	Plus							
	≤ 2000	52	-0.49 (-1.19, -0.04)	67.89%	49.49	0.0001		
	< 2000	3	-3.54 (-5.54, -1.55)	79.59%	57.28	0.0001	8.02	0.0001
	Follow up							
	< 5 weeks	22	-0.45 (-1.58, -0.04)	98.65%	88.99	0.001		
	5–10 weeks	11	-1.60 (-3.26, -0.05)	78.36%	56.11	0.001	1.86	0.600
	10–15 weeks	15	-0.36 (-1.06, -0.24)	96.07%	57.21	0.001		
	15–20 weeks	0	-	-	-	-		
	> 20	7	-1.87 (-3.61, -0.23)	69.56%	73.43	0.001		
	Type of ESWT							
	Radial	13	-0.49 (-1.27, -0.09)	88.99%	50.31	0.001		
	Focused	7	-1.48 (-3.10, -0.14)	87.03%	49.04	0.001	1.21	0.550
	Not determined	35	-0.55 (-1.57, 0.44)	91.34%	38.9	0.001		
	Type of comparisons							
	CSI	2	-1.19 (-3.55, -0.38)	77.12%	23.67	0.001		
	Exercise	5	-0.34 (-1.03, -0.03)	0.00%	1.22	0.56	6.20	0.403
	KT	6	0.57 (-2.19, 3.33)	89.04%	73.4	0.001		
	PBMT	4	0.05 (-0.40, 0.50)	56.39%	6.88	0.083		
	Placebo	25	-0.77 (-1.78, -0.25)	63.29%	13.79	0.001		
	US	10	-1.94 (-4.24, -0.36)	88.79%	40.67	0.001		
	WES	3	-0.21 (-0.49, -0.06)	0.00%	1.15	0.566		

CSI: corticosteroids injections, FoSW: Focused ESWT, RaSW: Radial ESWT, ESWT: Extracorporeal shock wave therapy, Autologous conditioned plasma: ACP; Platelet-rich plasma: PRP, LE: Lateral epicondylitis, Cryo-US: Cry- ultrasound, WES: Wrist extensor splint; Photo-biomodulation therapy (PBMT); RCT: Randomized controlled trials; CI: Confidence interval

### Mean pain in patients with CAT

**Fig. 4:**
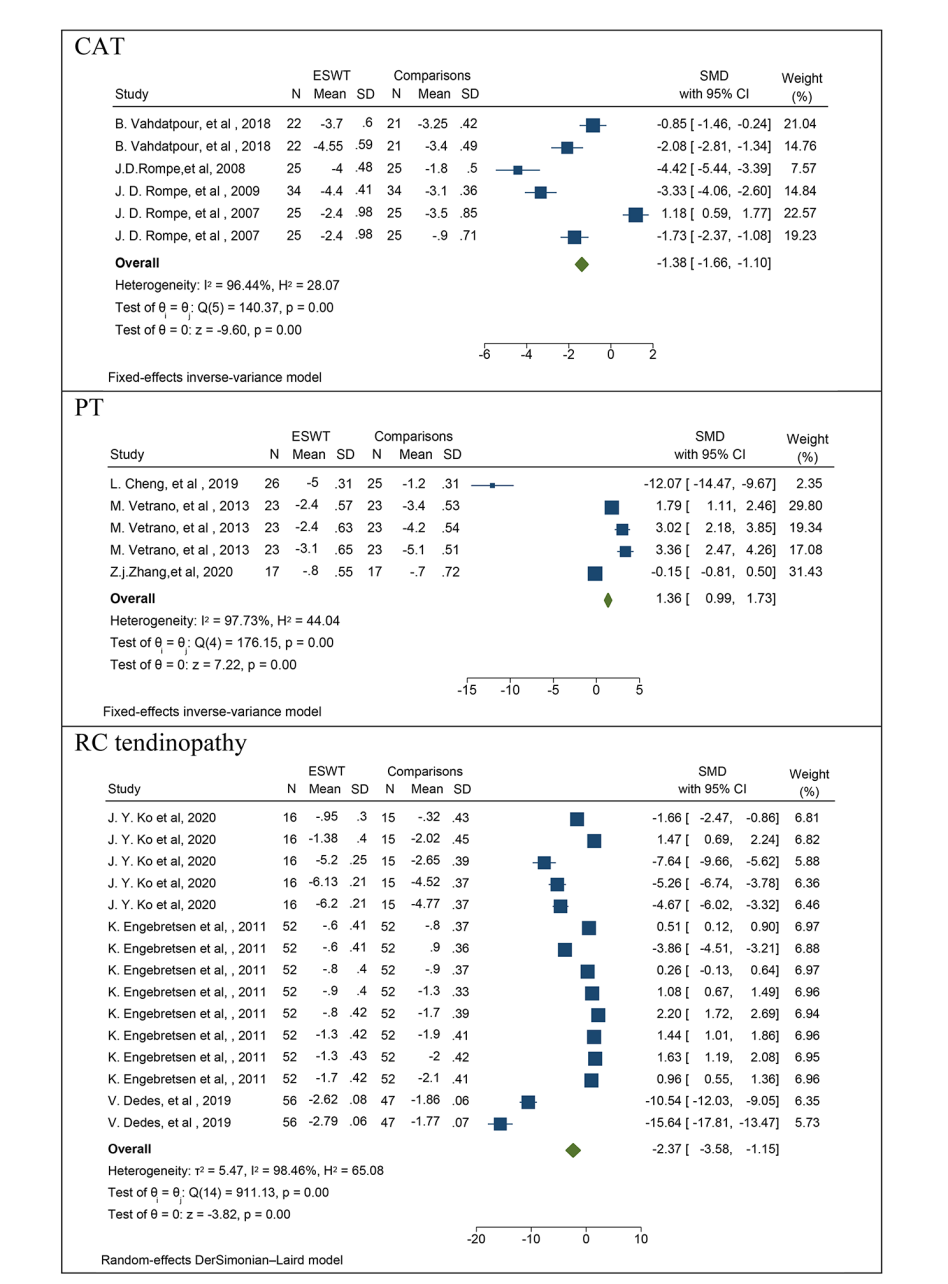
The forest plot displays the pooled standardized mean difference (SMD) for pain among patients with CAT, PT, RCL

In the analysis of studies examining the effect of ESWT on mean pain scores in patients with CAT, a total of 2 studies with 6 different effect sizes were included. The combined results of these studies, based on the reported effect sizes, indicated that the mean pain score in patients with CAT decreased by an average of 1.38 units when ESWT was utilized (SMD: -1.38, 95% CI: -1.66, -1.10; I2: 96.44%; P heterogeneity: 0.0001) (Fig. 4). Due to the limited number of studies available for analysis, subgroup analyses and funnel plots were not reported. However, publication bias was assessed using Egger’s test, which showed no evidence of publication bias (B: -1.02; SE: 0.99; P: 0.882).

### Mean pain in patients with PT

In the analysis of studies examining the effect of ESWT on mean pain scores in patients with PT, a total of 3 studies with 5 different effect sizes were included. The combined results of these studies, based on the reported effect sizes, indicated that the mean pain score in patients with PT increased by a mean of 1.36 units when ESWT was utilized (SMD: 1.36, 95% CI: 0.99, 1.73; I2: 97.73%; P heterogeneity: 0.0001) (Fig. 4). Due to the limited number of studies available for analysis, subgroup analyses and funnel plots were not reported. However, publication bias was assessed using Egger’s test, which showed no evidence of publication bias (B: 0.99; SE: 0.23; P: 0.540).

### Mean pain in patients with RC tendinopathy

To determine the effect of ESWT on the mean pain score in patients with RC tendinopathy, a total of 3 studies with 15 different effect sizes were analyzed. The combined results of these studies and the reported effect sizes indicated that the mean pain in patients with RC tendinopathy decreased by 2.37 units on mean when ESWT was used (SMD: -2.37, 95% CI: -3.58, -1.15; I2: 98.46%; P heterogeneity: 0.0001) (Fig. 4). Subgroup analyses and funnel plots were not reported due to the limited number of studies. Egger’s test was used to assess publication bias and the results showed no evidence of publication bias (B: -1.99; SE: 0.99; P: 0.495).

### Risk of bias results

**Fig. 5:**
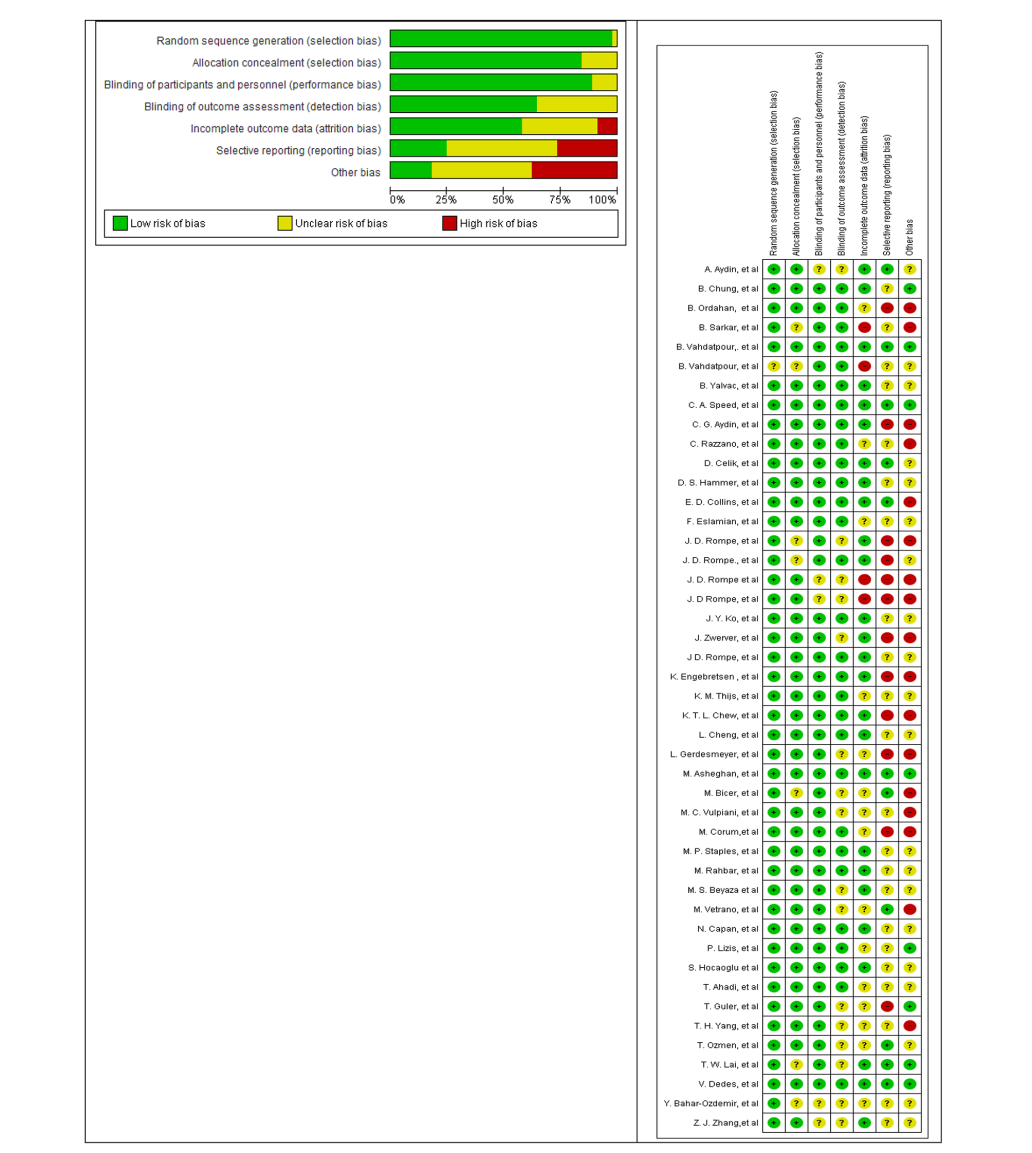
Risk of bias graph: review authors’ judgements about each risk of bias item presented as percentages across all included studies

The results of the quality assessment of the selected studies in this meta-analysis using the RoB2 showed that the majority of the selected studies were of sufficient quality to perform the meta-analysis. A small number of studies had a high risk of bias. In addition, some cases of bias, such as blinding of participants and personnel (performance bias), blinding of outcome assessment (detection bias), incomplete outcome data (attrition bias), and selection bias (reporting bias), were rated as “unclear” in the 6 items specified (Fig. 5).

## Discussion

In this meta-analysis, all clinical trials investigating the effects of ESWT on pain reduction in patients with different types of tendinopathies were evaluated. The overall findings of the meta-analysis demonstrated a significant reduction in pain with the use of ESWT. Furthermore, the meta-analysis revealed that FoSW was found to be more effective in reducing pain compared to RaSW. However, it is important to note that individualized decision-making is necessary when choosing between RaSW and FoSW treatments. Factors such as the specific type and location of the tendinopathy, patient characteristics, and treatment goals should be considered by clinicians when selecting the most appropriate treatment approach. Nowadays, ESWT is increasingly used in Orthopaedics, sports medicine, and other related fields to treat musculoskeletal injuries [109–111]. Although the mechanism of action of this intervention is not fully understood, its beneficial effects are likely to be related to micro-displacements. Several study results have shown that low-level shock waves from ESWT can induce various beneficial tissue responses and associated metabolic effects [32, 33, 109–113]. It is hypothesized that the utilization of focused shock waves may induce micro-trauma to avascular and hypo-vascular tissues, thereby stimulating the local release of growth factors and facilitating the recruitment of stem cells. Consequently, this process promotes vascular regeneration and subsequent tissue healing [114, 115]. The resulting changes from this process increase joint flexibility, provide long-term pain relief, and restore normal muscle tone [36, 116, 117].

To substantiate the effect of ESWT on pain reduction in patients with PF, previous research findings have demonstrated that ESWT accelerates vascular regeneration by promoting the release of growth factors and facilitating the mobilization of stem cells [118–120]. The results of previous studies in this area also support this finding, indicating a favorable and sustained effect of pain reduction with ESWT in patients with PF [118–122]. In the combination of studies and pooled estimate of the SMD regarding the effect of ESWT on mean pain in patients with PF, the percentage of heterogeneity was 77.36%, which is high but acceptable. The reason for this level of heterogeneity can be attributed to differences in the number of ESWT sessions, their intensity, baseline characteristics of patients such as the presence of other underlying diseases or other musculoskeletal disorders, BMI, age, etc. In this meta-analysis, subgroup analyses were performed based on important variables reported in selected clinical trials, and the results were reported. The reason for these subgroup analyses was to identify sources of heterogeneity. Subgroup analyses for the primary outcome were performed based on age, BMI, number of sessions, pulse intensity and frequency of the intervention, duration of patient follow-up after the intervention, and type of comparison group. The data suggests that ESWT shows greater effectiveness in treating PF in individuals over 30 years old with a BMI exceeding 25 compared to those with a lower BMI and younger age. This disparity in efficacy indicates potential differences in treatment response or levels of inflammation among these subgroups. Older individuals or those with a higher BMI may respond more positively to ESWT due to changes in tissue aging or other physiological factors. Moreover, these individuals might have increased inflammation in PF-affected areas, leading to more significant and successful pain relief with ESWT. Additional factors contributing to these variations include age-related physiological changes that support tissue repair and regeneration in older individuals, as well as obesity-related factors like heightened stress levels that ESWT could help alleviate, thereby reducing their discomfort [123–125]. Regarding the characteristics of ESWT, the results of the meta-analysis indicate that certain parameters contribute to its effectiveness in reducing pain in patients with PF. Specifically, a frequency of less than 10 Hz, a pulse intensity of less than 2000, and more than 5 treatment sessions were associated with increased effectiveness. These differences in effectiveness can be attributed to physiological variations among patients. However, in the case of a pulse intensity of less than 10, it can be argued that performing more sessions with a lower pulse intensity reduces the risk of damage to surrounding tissues and enhances the precision of targeting ESWT waves. Additionally, a frequency of less than 10 Hz may facilitate deeper penetration of shock waves and deeper stimulation in the treated area, ultimately resulting in a greater reduction in pain. Furthermore, the impact of ESWT on pain reduction demonstrated a larger effect size when compared to the placebo group than to other comparator groups. This difference is likely due to the phenomenon where even minimal improvements in pain perception are attributed to shock wave therapy, thus overshadowing the effects of previous interventions. Moreover, patient expectations and behaviors may also contribute to an amplified perception of ESWT efficacy. This supposition aligns with existing literature that highlights the effectiveness of ultrasound and other therapies, suggesting a similar underlying mechanism. It is important to consider these findings in the context of the meta-analysis and the included studies. Additionally, further research is necessary to explore the optimal parameters and protocols for ESWT in the treatment of PF and to gain a deeper understanding of the mechanisms underlying its effects [126–128].

The next outcome in this meta-analysis was the effect of ESWT on average pain in patients with LE. After combining selected studies to estimate the precise effect of ESWT on the crude average pain score in patients with LE, the results showed that, overall, the average pain in these patients decreased by 0.63 units based on the crude score of the VAS tool. While the diagnosis of LE is usually straightforward, its treatment presents several challenges [49, 129, 130].

The findings of the present meta-analysis indicate that the utilization of ESWT may yield positive outcomes in terms of pain reduction in patients with LE. The significance of this effect can be evaluated based on the examined CI. The 95% CI for the effect of ESWT on mean pain in patients with LE ranged from 1.11 to 0.16. The narrowness of this CI suggests the clinical significance or importance of the impact of ESWT on mean pain. Subgroup analysis based on the type of LE revealed that the effect of ESWT in reducing mean pain was more pronounced in patients with chronic LE compared to those with non-chronic LE. This disparity may be attributed to variations in characteristics or treatment response between the two types of LE. Chronic LE might possess distinct characteristics or underlying factors that make it more responsive to shockwave therapy, thereby resulting in greater improvement. This difference could stem from physiological or disease-related mechanisms. Moreover, the involved tissues and structures in the inflammatory and pain processes may differ in chronic LE, rendering them more receptive to shockwave therapy. Overall, these discrepancies may arise from distinct physiological or pathophysiological effects observed in the chronic and non-chronic LE groups. Further research is warranted to validate and elucidate these finding [131, 132].

In this meta-analysis, due to the availability of an adequate number of studies for LE and PF, more results were reported on the outcome of pain reduction in these two types of tendinopathies. However, among other types of tendinopathy examined in this meta-analysis, CAT can be mentioned. The results indicated that the use of ESWT can moderately reduce pain in individuals with this type of tendinopathy by 1.36 units. ESWT, by stimulating and stimulating the area of the tendon, improves blood flow and increases oxygen to the damaged tissues. It is also possible that it causes the breaking of calcium stones and scar tissue in the area of tendinopathy, which can facilitate the process of reconstructing damaged tissues [133, 134]. Via mechanical stimulation in the therapeutic mechanism, ESWT has the potential to enhance the expression of inflammation factors, promote tenocyte proliferation, and stimulate collagen synthesis, thereby facilitating the repair of damaged tendinous tissue and improving Achilles tendon function [126, 135]. Additionally, shock waves may have a beneficial effect on reducing local substance P levels [136] and damaging unmyelinated nerve fibers [137], ultimately leading to pain relief in CAT. The use of ESWT in reducing or managing pain in CAT patients, due to minimal invasive side effects, very safe benefits, and its economic advantages compared to other interventions, is gradually increasing. However, according to the study by Stania et al. [138], further investigations are needed in this area, considering factors such as the complexity of results and biological responses in CAT, as well as the wide variety of different ESWT algorithms [138].

The results from the meta-analysis reveal that ESWT significantly reduces pain levels in patients with RC tendinopathy, mirroring its effectiveness seen in CAT. Among RC tendinopathy patients treated with ESWT, there was an average pain decrease of 2.37 units. Several studies have affirmed the efficacy of ESWT in managing RC tendinopathy, leading to improvements in pain, functionality, and decreased calcification within the affected tendon. Compared to sham-ESWT or ultrasound-guided needling, ESWT emerges as a preferable treatment option. While the precise mechanisms underlying ESWT’s effectiveness in addressing RC tendinopathy are not fully elucidated, it is believed to involve stimulating the body’s natural healing processes, releasing growth factors, improving blood circulation, and mitigating inflammation within the affected tendon [139, 140]. Furthermore, ESWT demonstrates an analgesic effect, aiding in pain reduction among individuals with RC tendinopathy. However, according to the results of this meta-analysis, the use of ESWT in patients with PT had no significant impact on pain reduction. This finding contradicts the results of published studies regarding the effect of ESWT on average pain in PT patients. For example, a study conducted by Charles R [126]. and colleagues in 2023 demonstrated that ESWT reduces pain in patients with PT. Additionally, a study by Mani-Babu S [56]. and colleagues in 2015 emphasized that the use of ESWT can be effective in reducing pain in patients with tendinopathy, particularly those with PT. Generally, due to the lack of clinical guidelines for PT, a systematic review of the literature combining evidence on the effectiveness of ESWT compared to other interventions can enhance clinical decision-making in this regard. The results of the current meta-analysis may suggest the need for better and more accurate information regarding the use of ESWT in patients with PT, shaping the perspective that further research is required before considering ESWT as a treatment option for PT patients.

This meta-analysis represents a significant contribution to the field and is recognized for its comprehensive evaluation of the effects of ESWT on patients with tendinopathy. This recognition is based on the thorough review and analysis of all outcomes and different types of tendinopathy within this study. In addition, subgroup analyses based on key influencing variables were performed to elucidate the effect of ESWT in patients with tendinopathy, providing valuable insights. Furthermore, this study meticulously reported all sources of heterogeneity and performed desired analyses such as meta-regression and publication bias analysis. The sample size in the selected studies and the number of selected studies in this review are much larger than the systematic reviews and meta-analyses published to date [52, 54, 141, 142]. With these explanations, it can be said that the results of the present meta-analysis can be a valuable source for updating therapeutic and care guidelines related to different types of tendinopathy. Furthermore, the results of the present meta-analysis indicate the superior and effective impact of minimally invasive or non-invasive approaches, particularly ESWT, in reducing pain in various patients with tendinopathy. This reduces the significant need for invasive methods such as high-risk surgery or other orthopedic procedures.

Limitations of this study include the lack of subgroup analysis based on other important variables, such as the duration of pain, the presence or absence of other underlying diseases or other musculoskeletal disorders, which was not performed due to the lack of reporting results on the effect of ESWT on average pain in patients with various types of tendinopathy in the initial selected studies. Also, the lack of reporting subgroup analyses to determine the effect of ESWT on average pain in patients with RC tendinopathy, CAT, and PT was due to the limited number of published studies on this matter worldwide.

## Conclusion

The results of this meta-analysis show that the use of ESWT can have a significant impact on reducing mean pain in patients with different types of tendinopathy. In addition, the results confirm that the effectiveness of ESWT in these patients is greater when applied at lower intensities and for longer durations, taking into account the age and BMI of the patients. Therefore, in the primary treatment and care of these patients, an accurate assessment of their condition and consideration of the benefits and possibilities of using ESWT is essential. These results suggest that health policy makers and health care providers should focus on non-invasive programmers and treatments for patients with tendinopathy.

### Electronic supplementary material

Below is the link to the electronic supplementary material.

Supplementary Material 1

## Data Availability

The datasets used and analyzed during the current study are available from the corresponding author upon reasonable request.

## Abbreviations

ESWT: Extracorporeal shock wave therapy
RaSW: Radial extracorporeal shock wave therapy
FoSW: Focused extracorporeal shock wave therapy
CENTRAL: Cochrane central register of controlled trials
RCT: Randomized control trials
RoB2: The Cochrane risk-of-bias tool for randomized trials
PF: Plantar fasciitis
LE: Lateral epicondylitis
CAT: Chronic Achilles tendinopathy
SMD: Standardized mean difference
NSAIDs: Nonsteroidal anti-inflammatory drugs
PRISMA: The Preferred Reporting Items for Systematic Reviews and Meta-Analyses
RC tendinopathy: Rotator cuff tendinopathy
PT: Patellar tendinopathy
CSI: Corticosteroids injections
NRSS: Numerical Rating Scale Scores
KT: Kinesio taping
NIN: Noninvasive Interactive Neurostimulation
ACP: Autologous Conditioned Plasma
PRP: Platelet-rich plasma
Cryo-US: Cry-ultrasound
WES: Wrist extensor splint
